# Comparison of the perioperative outcomes of robotic vs. open distal pancreatectomy: a meta-analysis of propensity-score-matched studies

**DOI:** 10.3389/fsurg.2025.1611773

**Published:** 2025-12-04

**Authors:** Junjie Wang, Yuanjun Liu, Yakun Wu

**Affiliations:** Department of Hepatobiliary Surgery, Suining Central Hospital, Suining, Sichuan, China

**Keywords:** robotic distal pancreatectomy, open distal pancreatectomy, mortality, morbidity, meta-analysis

## Abstract

**Objective:**

Robotic distal pancreatectomy (RDP) is considered to offer certain advantages over traditional open distal pancreatectomy (ODP); however, high-quality evidence remains limited. This meta-analysis aimed to compare perioperative outcomes between RDP and ODP using data from propensity-score–matched studies.

**Methods:**

A systematic literature search was performed using the PubMed, Cochrane Library, Embase, and Web of Science databases for studies comparing RDP and ODP. Odds ratios (ORs) and mean differences (MDs) with 95% confidence intervals (CIs) were calculated.

**Results:**

Seven studies with 1,526 patients were included (RDP group: 722 patients; ODP group: 804 patients). Compared with ODP, RDP was associated with a shorter hospital stay (MD −3.11 days; 95% CI, −4.45, −1.77), reduced blood loss (MD −163.38 mL; 95% CI, −212.08, −114.68), higher spleen preservation rates (OR 2.36, 95% CI, 1.06, 5.24) and lower surgical site infection (SSI) rates (OR 0.47, 95% CI 0.29, 0.76). No significant differences were found in 90-day mortality, overall morbidity, major complications, operative time, reoperation rates, postoperative pancreatic fistula, number of harvested lymph nodes, and R0 resection rates.

**Conclusions:**

This meta-analysis suggests that RDP may have potential advantages over ODP, including reduced blood loss, shorter hospitalization, higher spleen preservation, and lower SSI rates. These potential benefits warrant confirmation in future randomized controlled trials.

**Systematic Review Registration:**

https://www.crd.york.ac.uk/PROSPERO/view/CRD420251031280, PROSPERO CRD420251031280.

## 1 Introduction

Pancreatic surgery remains one of the most technically demanding abdominal procedures, associated with high postoperative morbidity (1, 2). Although advances in surgical techniques and perioperative management have markedly reduced postoperative mortality in high-volume centers, the incidence of complications after distal pancreatectomy (DP) remains as high as 40%–60% (3).

Compared with open surgery, minimally invasive approaches offer several advantages, including reduced surgical trauma, less intraoperative blood loss, and faster recovery, which may translate into improved perioperative outcomes (4, 5). Since Gagner et al. first reported minimally invasive DP in 1996, laparoscopic distal pancreatectomy (LDP) has gained increasing acceptance among pancreatic surgeons (6). However, conventional laparoscopy is limited by reduced instrument dexterity, two-dimensional visualization, and a steep learning curve (7). In contrast, robotic surgery maintains the advantages of minimally invasive techniques while providing three-dimensional visualization, enhanced instrument flexibility, tremor filtration, and a shorter learning curve (6, 7). Several cohort studies have compared robotic distal pancreatectomy (RDP) and open distal pancreatectomy (ODP) in terms of surgical outcomes (8–10). However, due to potential differences in baseline characteristics between study groups, results from these observational studies remain inconsistent and inconclusive (11). There is still a lack of high-quality evidence to confirm the advantages of robotic surgery in DP. To date, no randomized controlled trials (RCTs) have directly compared the efficacy of RDP and ODP. Propensity score matching (PSM) is a robust statistical approach that minimizes confounding by balancing baseline variables between groups, thereby reducing selection bias in observational studies (11, 12). Well-designed PSM studies have been shown to provide evidence comparable to that of RCTs (12, 13). In recent years, several PSM studies (2, 6, 14) have examined RDP vs. ODP, yet their findings remain heterogeneous.

Therefore, to provide more robust and high-quality evidence regarding the role of robotic surgery in DP, we conducted a comprehensive meta-analysis including only PSM studies to compare the short-term outcomes of RDP and ODP.

## 2 Methods

### 2.1 Search strategy

This study was conducted in accordance with the Preferred Reporting Items for Systematic Reviews and Meta-Analyses (PRISMA) (15). The study protocol was registered with the PROSPERO database.

Two authors (JW and YL) independently conducted a comprehensive literature search using the EMBASE, Web of Science, PubMed, and Cochrane Library databases to identify potential studies published before February 25, 2025. The detailed search strategy is provided in Table 1. In addition, we checked the reference lists of the identified articles and related reviews to identify further eligible studies. No language restrictions were applied.

**Table 1:** Electronic search strategy.

Database	Search term (published up to February 25, 2025)	Number
PubMed	((Da Vinci[Title/Abstract]) OR (Robot*[Title/Abstract]) OR (Robot-assisted[Title/Abstract]) OR (Robotic-assisted[Title/Abstract])) AND ((distal pancreatectomy[Title/Abstract]) OR (pancreatectomy[Title/Abstract]))	633
Embase	(distal pancreatectomy OR pancreatectomy).ab,kw,ti. AND (Da Vinci OR Robot* OR Robot-assisted OR Robotic-assisted).ab,kw,ti.	1,072
Cochrane Library Trials	[(distal pancreatectomy OR pancreatectomy):ti,ab,kw] AND [(Da Vinci) OR Robot* OR Robot-assisted OR Robotic-assisted:ti,ab,kw]	56
Web of Science	(TS = [(Da Vinci) OR (Robot*) OR (Robot-assisted) OR (Robotic-assisted)]) AND TS = [(distal pancreatectomy) OR (pancreatectomy)]	689

### 2.2 Study selection

The inclusion criteria were: (1) Patient: adult patients who were undergoing DP; (2) Intervention: RDP; (3) Comparison: ODP; (4) Outcomes: Primary outcomes included 90-day mortality, overall complication, major complication, and length of stay. Secondary outcomes included blood loss, operative duration, spleen preservation, reoperation, postoperative pancreatic fistula (POPF), surgical site infection (SSI), number of harvested lymph nodes, and R1 resection; (5) Study type: PSM studies.

Reviews, letters, case reports, conference abstracts, single-arm studies, animal studies, and repeated publications were excluded.

### 2.3 Data extraction

The following data were extracted independently by two authors (JW and YL): author name, year of publication, country, study design, study population (sample size, sex, age, and body mass index), intraoperative information (blood loss, operative duration, and spleen preservation) and short-term outcomes. When data of interest were unavailable, the corresponding author was contacted to obtain the necessary data.

### 2.4 Quality assessment

The quality assessment was conducted independently by two authors (JW and YL) using the Newcastle-Ottawa Scale (NOS), which assigns a score on a 9-point scale. A score of ≥7 indicates high quality, and scores of 5–6 indicate moderate quality. Any discrepancies were resolved through discussion, with intervention by a third author (YW) whenever necessary.

### 2.5 Statistical analysis

In this study, statistical analyses were performed using the Review Manager software (version 5.3). Odds ratios (ORs) with corresponding 95% confidence intervals (CI) were calculated for qualitative variables and the mean difference (MD) for quantitative data. The I² statistic was used to quantify heterogeneity. A random-effects model was used if *I*² > 50%; otherwise, a fixed-effects model was employed (16). To explore the robustness of the results, we adopted the one-study exclusion method to evaluate the impact of each study on the overall effect size. Publication bias was assessed using Egger's test and funnel plot (if the number of included studies was more than 10) for primary outcomes. Statistical significance was set at *p* < 0.05.

## 3 Results

### 3.1 Study selection

**Figure 1:**
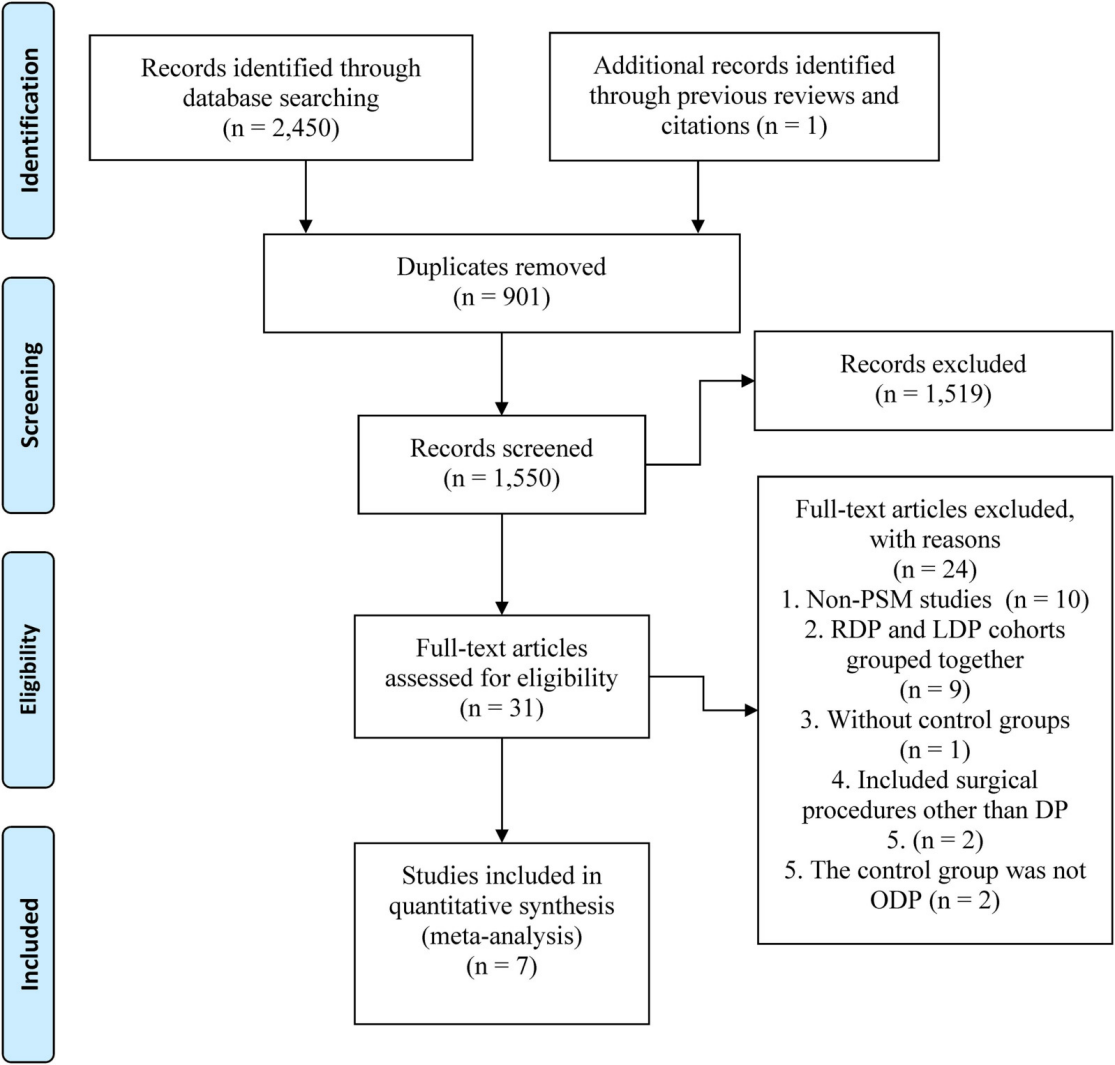
The PRISMA flowchart.

The search yielded 2,451 records, of which 901 duplicates were excluded. After reviewing titles and abstracts, 1,519 studies were excluded, and the full-texts of the remaining 31 articles were evaluated. Finally, 7 studies (1–3, 5, 6, 14, 17) were included in the final analysis (Figure 1).

### 3.2 Study characteristics and quality assessment

Table 2 summarizes the characteristics of the 7 included studies (1–3, 5, 6, 14, 17). The studies were published between 2019 and 2024 and included 1,526 patients (RDP group: 722 patients; ODP group: 804 patients). The included patients were mainly from the United States, China, Italy, and Spain. All studies were considered of high quality, achieving a score of ≥7 based on the NOS. The relevant details of the PSM methods used in each included study are summarized in Table 3.

**Table 2:** Characteristics of the included studies.

First author, year	Country	Study period	Male	Study design	Age	BMI	Sample size	NOS
Ielpo, et al.(1)	Spain	2010–2017	RDP: 16	RCS, PSM	RDP: 59.7 (10.5)	RDP: 24.1 (4.5)	RDP: 28	8/9
			ODP: 15		ODP: 62.5 (11.3)	ODP: 23.4 (4.1)	ODP: 28	
Weng, et al. (17)	China	2012–2019	RDP: 69	RCS, PSM	RDP: 50.4 (15.5)	RDP: 23.2 (3.5)	RDP: 219	9/9
			ODP: 86		ODP: 51.0 (14.6)	ODP: 23.2 (3.9)	ODP: 219	
Chen, et al. (11)	China	2011–2018	RDP: NA	RCS, PSM	RDP: NA	RDP: NA	RDP: 130	8/9
			ODP: NA		ODP: NA	ODP: NA	ODP: 130	
Klompmaker, et al. (5)	USA	2006–2017	RDP: NA	RCS, PSM	RDP: NA	RDP: NA	RDP: 79	8/9
			ODP: NA		ODP: NA	ODP: NA	ODP: 79	
Magistri, et al. (14)	USA	NA	RDP: 33	PCS, PSM	RDP: 63 (19–81)	RDP: 25 (17–34)	RDP: 82	9/9
			ODP: 66		ODP: 63 (18–85)	ODP: 25 (17–38)	ODP: 164	
Song, et al. (6)	China	2017–2021	RDP: 97	RCS, PSM	RDP: 60 (53, 66)	RDP: 23.51 (21.6–25.68)	RDP: 159	8/9
			ODP: 97		ODP: 60 (54, 67)	ODP: 23.05 (20.88, 25.42)	ODP: 159	
Bencini, et al. (2)	Italy	2013–2022	RDP: 10	RCS, PSM	RDP: 69 (27–81)	RDP: 28 (18–34)	RDP: 25	7/9
			ODP: 10		ODP: 67 (48–78)	ODP: 25 (18–34)	ODP: 25	

ODP, open distal pancreatectomy; NA, not available; PCS, prospective cohort study; PSM, propensity score matching; RCS, retrospective cohort study; RDP, robotic distal pancreatectomy.

**Table 3:** Details of the propensity score–matched methods.

First author, year	Matching variables	Ratios (ODP: RDP)	Balance diagnostics
Ielpo, et al. (1)	Age, gender, BMI, ASA score, malignancy, and tumor size.	1:1	NA
Weng, et al. (17)	Age, sex, BMI, ALB level, previous abdominal surgery history, ASA physical status, CA 19-9 level, and PV/SMV abutment, together with the variations in tumor size, pathological type and tumor location	1:1	NA
Chen, et al. (11)	Age, sex, BMI, ASA scores, comorbidities, albumin, NLR, CA19-9, major vessel resection, transection planes, transection methods, nerve plexus invasion, tumor size, and tumor differentiation	1:1	NA
Klompmaker, et al. (5)	Age, sex, BMI, Charlson comorbidity index, history ofpelvic/abdominal surgery, ASA-classification, tumor size, working diagnosis, and pancreatic texture (soft vs not soft).	1:1	SMD
Magistri, et al. (14)	Age, ASA score, BMI, final pathology, and TNM (Tumour, Node, Metastasis) staging system VIII	2:1	NA
Song, et al. (6)	Age, sex, BMI, ASA score, AJCC staging for PDAC, tumour diameter and tumour differentiation	1:1	NA
Bencini, et al. (2)	ASA score, CCI index, and neoadjuvant chemotherapy	1:1	NA

ASA, American society of anesthesiology; BMI, body mass index; ODP, open distal pancreatectomy; NA, not available; RDP, robotic distal pancreatectomy; SMD, standardized mean difference.

### 3.3 Meta-analysis

#### 3.3.1 90-day mortality

**Figure 2:**
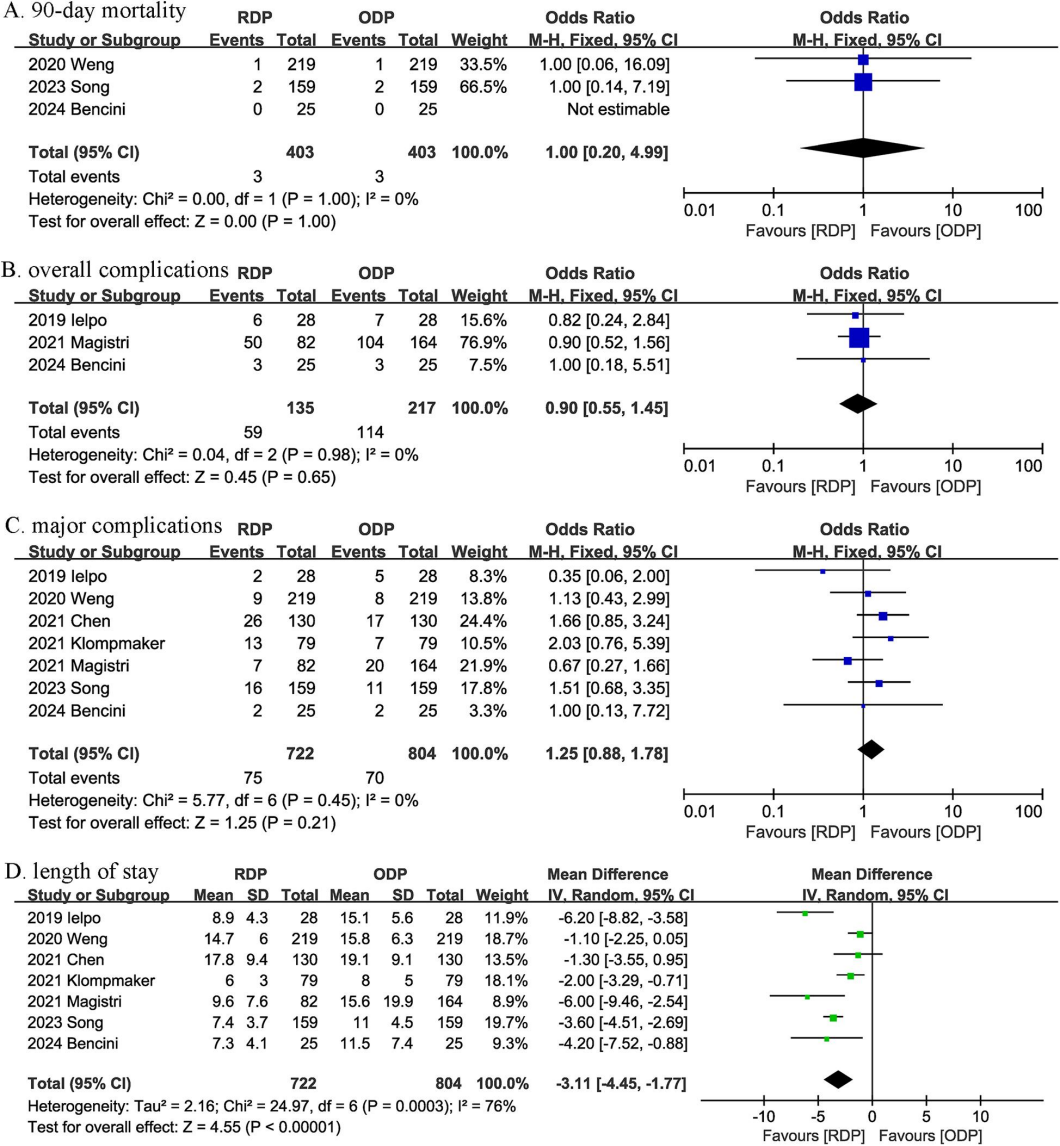
Comparison of primary outcomes between the two groups. **(A)** 90-day mortality, **(B)** overall complications, **(C)** major complications, and **(D)** length of stay.

Three studies reported data on 90-day mortality. The combined results of the 3 studies showed that there was no significant difference between the RDP group and the ODP group regarding this outcome with low heterogeneity (OR 1.00, 95% CI 0.20, 4.99; Heterogeneity: *I*^2^ = 0%, *P* = 1.00) (Figure. 2A) (Table 4).

**Table 4:** Outcomes of interest RDP vs. ODP.

Outcomes	No. of studies	Events for RDP	Events for ODP	Effect size	95% CI	*P*	*I*^2^ (%)
90-day Mortality	3	3/403	3/403	1.00	0.20, 4.99	1.00	0
Overall complications	3	59/135	114/217	0.90	0.55, 1.45	0.65	0
Major complications	7	75/722	70/804	1.25	0.88, 1.78	0.21	0
Length of stay	7	-	-	−3.11	−4.45, −1.77	<0.00001	76
Blood loss	6	-	-	−163.38	−212.08, −114.68	<0.00001	87
Operation time	7	-	-	7.35	−25.37, 40.06	0.66	96
R0 resection	5	583/618	649/700	1.26	0.79, 2.00	0.33	41
Number of lymph nodes harvested	3	-	-	2.34	−3.05, 7.73	0.39	57
Postoperative pancreatic fistula	7	125/722	147/804	0.97	0.74, 1.26	0.80	0
Surgical site infection	3	29/367	56/367	0.47	0.29, 0.76	0.002	40
Spleen preservation rate	4	161/354	82/436	2.36	1.06, 5.24	0.04	66
Reoperation	5	16/567	15/649	1.31	0.66, 2.62	0.44	0

#### 3.3.2 Morbidity

Overall complication was reported in 3 studies. The pooled results suggested that the overall complication rate in the RDP group was comparable to that in ODP group (OR 0.90, 95% CI 0.55, 1.45, *P* = 0.65; *I*^2^ = 0%, *P* = 0.98) (Figure 2B).

#### 3.3.3 Major complications

Combined data from 7 studies showed that the rates of major complications were comparable between the RDP and ODP groups (OR 1.25, 95% CI 0.88, 1.78; Heterogeneity: *I*^2^ = 0%, *P* = 0.45) (Figure 2C).

#### 3.3.4 Length of stay

The length of the hospital stay was reported in 7 studies. According to the results of this meta-analysis, RDP significantly reduced the length of hospital stay (MD, −3.11 days; 95% CI, −4.45, −1.77, < 0.00001) (Figure 2D).

#### 3.3.5 Blood loss

**Figure 3:**
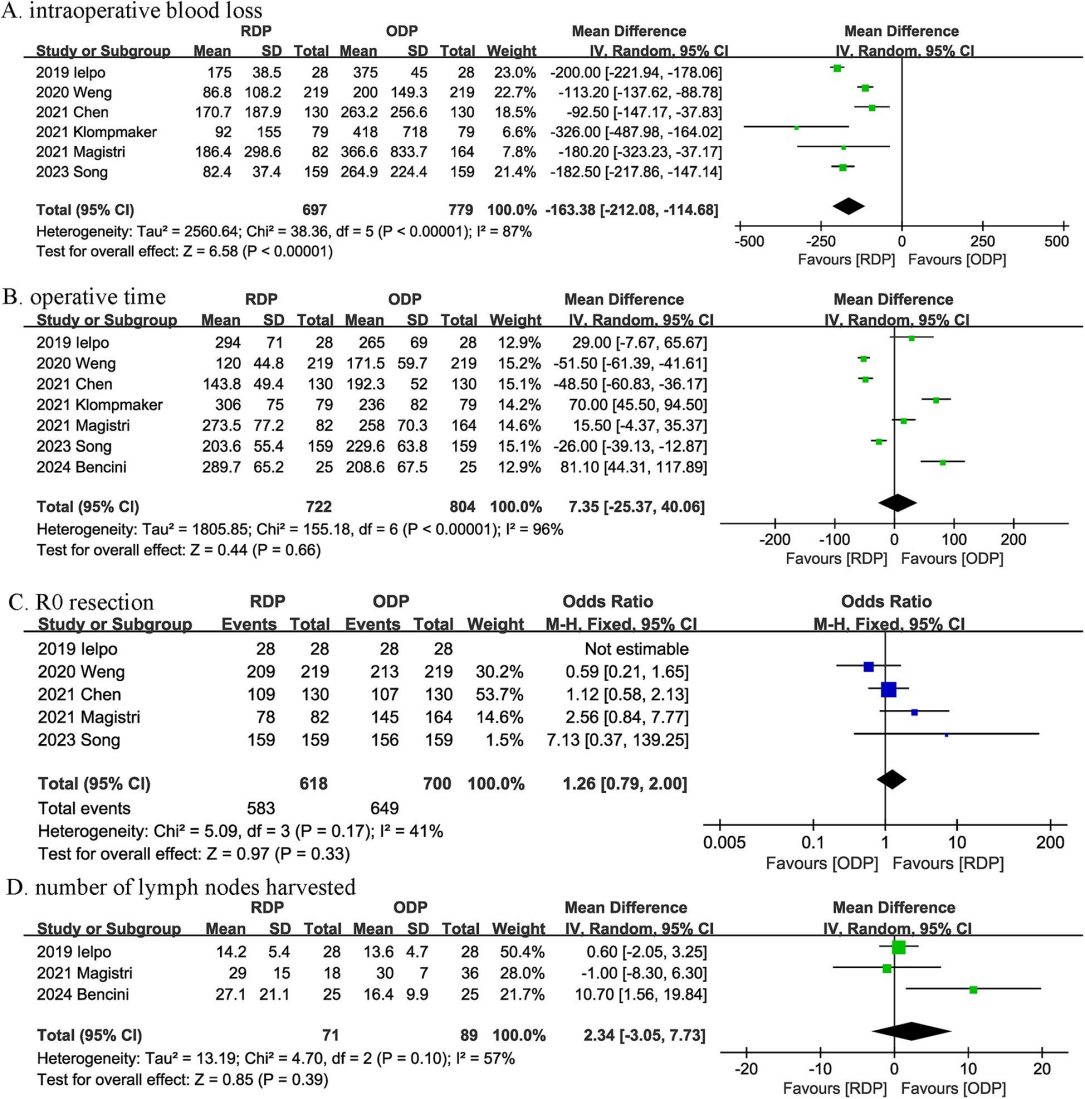
Comparison of secondary outcomes between the two groups. **(A)** intraoperative blood loss, **(B)** operative time, **(C)** R0 resection, and **(D)** number of lymph nodes harvested.

Six studies provided information on intraoperative blood loss. The combined results showed that RDP significantly reduced the amount of intraoperative blood loss (MD, −163.38 mL; 95% CI, −212.08, −114.68, <0.00001; *I*^2^ = 87%) (Figure 3A).

#### 3.3.6 Operation time

The operation time was reported in 7 trials. The combined results showed that the operation time was similar between the RDP group and the ODP group (MD, 7.35 min; 95% CI, −25.37, 40.06, *P* = 0.66) (Figure 3B).

#### 3.3.7 R0 resection

Five studies reported R0 resection, and the combined effect size suggested that the R0 resection rates were comparable between the two groups (OR 1.26, 95% CI 0.79, 2.00, *P* = 0.33; *I*^2^ = 41%) (Figure 3C).

#### 3.3.8 Number of lymph nodes harvested

Three trials reported the number of lymph nodes harvested, and no significant difference was observed between the groups (MD, 2.34; 95% CI, −3.05, 7.73, *P* = 0.39; *I*^2^ = 57%) (Figure 3D).

#### 3.3.9 Postoperative pancreatic fistula

**Figure 4:**
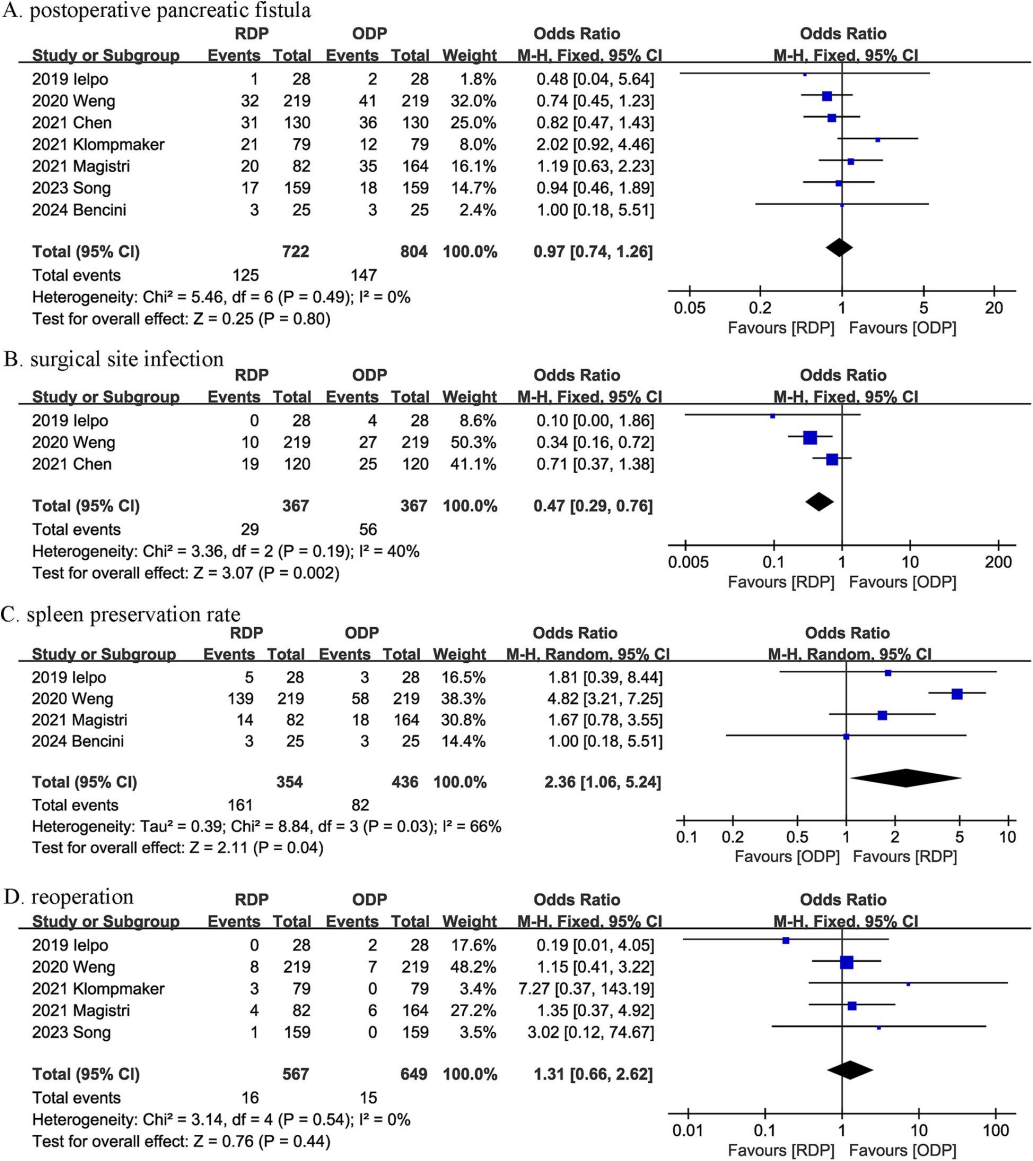
Comparison of secondary outcomes between the two groups. **(A)** postoperative pancreatic fistula, **(B)** surgical site infection, **(C)** spleen preservation rate, and **(D)** reoperation.

Seven studies reported the POPF. There was no significant difference in the incidence of POPF (OR 0.97, 95% CI 0.74, 1.26, *P* = 0.80) (Figure 4A) between the RDP and ODP groups.

#### 3.3.10 Surgical site infection

Three studies reported SSI. Compared with ODP, RDP significantly reduced SSI rate (OR 0.47, 95% CI 0.29, 0.76, *P* = 0.002), and the heterogeneity between studies was low (*I*^2^ = 40%, *P* = 0.19) (Figure 4B).

#### 3.3.11 Spleen preservation rate

Spleen preservation rate was evaluated in 4 studies, and the pooled results showed RDP significantly improved the splenic preservation rate (OR 2.36, 95% CI 1.06, 5.24; heterogeneity: *I*^2^ = 66%, *P* = 0.03) (Figure 4C).

#### 3.3.12 Reoperation

Reoperation was reported in 5 studies, and there was no significant difference in reoperation rates (OR 1.31, 95% CI 0.66, 2.62, *P* = 0.44) (Figure 4D) between the two groups.

### 3.4 Publication bias and sensitivity analysis

According to the Egger tests, and no significant publication bias was observed for overall complication (*P* = 0.651), major complications (*P* = 0.124), and length of stay (*P* = 0.258). Sensitivity analysis showed that no single study affected the overall effect size of the length of stay, 90-day mortality, overall complication, major complications, reoperation, operation time, blood loss, number of lymph nodes harvested, or R0 resection. The sensitivity analysis suggested that the total effect size of SSI changed significantly when the study by Weng et al. (17) (OR 0.61, 95% CI 0.32, 1.14, *P* = 0.12; *I*^2^ = 42%) was eliminated. The sensitivity analysis suggested that the total effect size of spleen preservation rate changed significantly when the study by lelpo et al. (1) (OR 2.42, 95% CI 0.94, 6.27, *P* = 0.07; *I*^2^ = 75%) or Weng et al. (17) (OR 1.58, 95% CI 0.84, 2.96, *P* = 0.16; *I*^2^ = 0%) was eliminated.

## 4 Discussion

Based on data from 1,526 patients across seven PSM studies, our meta-analysis demonstrated that RDP significantly reduced intraoperative blood loss, improved spleen preservation, lowered SSI rates, and shortened hospital stays compared with conventional ODP. In contrast, no significant differences were observed in 90-day mortality or morbidity. These findings provide important clinical evidence that RDP is not inferior to ODP in short-term safety and may offer additional perioperative benefits, assisting surgeons in selecting the optimal surgical approach.

In recent years, there has been increasing evidence that minimally invasive surgery, especially robotic surgery, can reduce surgical trauma and enhance postoperative recovery during major abdominal procedures such as colorectal surgery and gastrectomy (18–20). A RCT conducted by de Rooij et al. demonstrated that minimally invasive distal pancreatectomy (MIDP) promotes faster functional recovery and leads to improved quality of life compared with ODP (21). A meta-analysis by Manara et al. showed that robotic total gastrectomy significantly reduced intraoperative blood loss, time to first flatus, time to first ambulation, time to first liquid diet resumption, and hospital stay compared to open total gastrectomy (20). In addition, the study of Song et al. showed that robotic surgery could reduce the incidence of delayed gastric emptying (6). These improvements in postoperative recovery may contribute to shorter hospital stays, consistent with our finding that RDP was associated with a significantly shorter length of stay than ODP. A recent non-PSM study by Zhou et al. (9) also demonstrated that, compared with the conventional ODP group, the RDP group had less intraoperative blood loss, reduced transfusion requirements, and a shorter hospital stay. Postoperative complications are key indicators for evaluating the safety of surgical approaches, as they not only prolong hospitalization but also increase healthcare costs (22, 23). Weinberg et al. reported that patients with minor complications (Clavien–Dindo < III) experienced a median 17.1% increase in hospitalization costs, while those with major complications faced a 252% increase (23). Our study showed that the overall complication and major complication rates for RDP and ODP were comparable. The two groups were also comparable in terms of mortality. This is consistent with the results of the previous meta-analysis by Zhou et al. (7). Moreover, two RCTs have demonstrated that the postoperative complication rate of MIDP is comparable to that of ODP (21, 24). POPF remains one of the most common and clinically significant complications following DP (25). Reported incidences range from 24%–38% for all grades and 13%–17% for clinically relevant POPF (grade B and C) (25–27). The mortality of grade C POPF is as high as 30% (12). Considering the clinical value of POPF, we only analyzed the differences between the two surgical approaches in terms of clinically relevant POPF. Our study showed that the incidence of POPF in the robotic group (17.3%) was comparable to that in the open group (18.3%). SSI is the main cause of postoperative morbidity after major abdominal surgery. SSI greatly increases the financial burden and may require additional diagnostic tests and treatment (28). Several studies have suggested that robotic surgery may lower SSI risk (29, 30), likely due to smaller incisions and reduced tissue trauma (7). Our findings confirm the advantage of robotic surgery in decreasing SSI rates.

Spleen preservation is a key goal in DP for benign and low-grade malignant diseases (31, 32). However, maintaining splenic vasculature remains technically challenging during ODP (33). Robotic systems, with enhanced three-dimensional visualization, wristed instruments, and tremor reduction, facilitate these delicate dissections (7, 12). Several meta-analyses (34, 35) have shown that robotic surgery significantly improves spleen preservation compared to laparoscopic surgery. Our findings further confirm the benefits of RDP in preserving the spleen.

Complete tumor resection and adequate lymph node dissection are critical for the effective treatment of pancreatic tumors (36). Howard et al. demonstrated that R0 resection is associated with improved long-term survival (36). Furthermore, sufficient lymph node harvest is essential for accurate staging and prognosis (37). The number of lymph nodes acquired was an independent prognostic factor for patients. Wang et al. (37) reported that a minimum of 19 lymph nodes should be examined to ensure adequate staging in patients undergoing DP for pancreatic cancer. In our study, RDP and ODP were comparable in both R0 resection rates and the number of lymph nodes retrieved, consistent with the findings of Zhou et al. (7). Furthermore, a recent non-PSM study by Kamarajah et al. also demonstrated that there were no significant differences among RDP, LDP, and ODP in terms of R0 resection rates and the number of lymph nodes retrieved (38).

The high cost remains a major drawback of the robotic approach, which may be partly attributed to the additional instruments required for robotic surgery (2). Several retrospective studies (39, 40) have demonstrated that RDP is associated with significantly higher hospitalization costs compared with LDP. In contrast, RDP has been shown to reduce hospital costs relative to ODP (41, 42). With the increasing global adoption and technological maturation of robotic systems, the associated costs are expected to decline over time (39). However, because cost-effectiveness data were lacking in the included studies, further investigations are warranted to comprehensively assess the economic value of the robotic approach.

Our research has the following advantages. On the one hand, we conducted a comprehensive literature search, minimizing potential selection bias. On the other hand, we set strict inclusion criteria and included only PSM studies, enhancing the reliability of our results.

There are some limitations to this meta-analysis. First, the meta-analysis included a limited number of studies, some with small sample sizes. Second, all included studies were cohort studies, as no randomized controlled trials were available. Third, some outcomes (e.g., blood loss, length of hospital stay, operation time, number of lymph nodes harvested, and spleen preservation rate) exhibited high heterogeneity, which may be related to differences in surgeon experience, institutional volume, geographic region, and patient characteristics. In addition, the learning curve may be a critical factor influencing surgical outcomes. Chen et al. (3) reported that surgical performance could improve after overcoming the learning curve of RDP. However, even in high-volume centers, the learning curve for MIDP remains considerably long, requiring approximately 85 cases to achieve proficiency (43). Therefore, mastering MIDP still demands substantial practical experience. Due to the limited number of included studies, subgroup analyses could not be performed. Sensitivity analyses indicated that the results for blood loss, length of hospital stay, operation time, and number of lymph nodes harvested were robust. However, the spleen preservation rate was less stable, and further studies are needed to clarify this outcome. The heterogeneity of patient populations (benign lesions vs. malignant tumors) may influence the study results. Among the included studies, two (3, 6) enrolled only patients with malignant tumors, while five (1, 2, 5, 14, 17) included both benign and malignant cases. However, none provided separate analyses for benign and malignant cohorts. Therefore, we were unable to specifically evaluate the safety and efficacy of RDP in benign vs. malignant disease. Future studies are needed to investigate the outcomes of RDP in more defined patient populations. Apart from robotic surgery, laparoscopy represents another minimally invasive approach. However, the lack of comparative studies involving laparoscopic surgery limits the generalizability of our findings to broader clinical practice. Finally, although our findings suggest potential short-term benefits of RDP, the lack of long-term oncologic outcome data remains a major limitation. Further studies are warranted to comprehensively evaluate its long-term efficacy and oncologic safety.

In conclusion, this meta-analysis indicates that RDP achieves comparable short-term outcomes to ODP while significantly reducing intraoperative blood loss, enhancing splenic preservation, and lowering both SSI rates and length of hospital stay. Given that these findings are derived from non-RCTs, future high-quality RCTs with longer follow-up are warranted to further validate the potential benefits of RDP.

## Data Availability

The original contributions presented in the study are included in the article/Supplementary Material, further inquiries can be directed to the corresponding author.

## References

[1] Ielpo B Caruso R Duran H Diaz E Fabra I Malavé L Robotic vs. standard open pancreatectomy: a propensity score-matched analysis comparison. Updates Surg. (2019) 71(1):137–44. 10.1007/s13304-018-0529-1 29582359

[2] Bencini L Moraldi L Miceli E Risaliti M Tofani L Buccianti S Robotic vs. open distal pancreatectomy: a propensity score matching analysis. Int J Med Robot. (2024) 20(6):e70025. 10.1002/rcs.70025 39692257

[3] Chen H Shen Z Ying X Weng Y Jiang Y Chen H Robotic distal pancreatectomy reduces pancreatic fistula in patients without visceral obesity as compared to open distal pancreatectomy: a propensity score matching retrospective cohort study. Int J Surg. (2021) 90:105960. 10.1016/j.ijsu.2021.105960 33989824

[4] Tang G Zhang J Zhang L Xia L Chen R Zhou R. Postoperative complications and surgical outcomes of robotic vs. laparoscopic pancreaticoduodenectomy: a meta-analysis of propensity-score-matched studies. Int J Surg. (2025) 111(2):2257–72. 10.1097/js9.0000000000002196 39715160

[5] Klompmaker S van der Vliet WJ Thoolen SJ Ore AS Verkoulen K Solis-Velasco M Procedure-specific training for robot-assisted distal pancreatectomy. Ann Surg. (2021) 274(1):e18–27. 10.1097/sla.0000000000003291 30946088

[6] Song Y Zou W Gao Y Zhao Z Yin Z Xiao C Short- and long-term outcomes of robotic vs. open radical antegrade modular pancreatosplenectomy: a retrospective propensity score-matched cohort study. Surg Endosc. (2024) 38(3):1316–28. 10.1007/s00464-023-10635-4 38110793

[7] Zhou J Lv Z Zou H Xiong L Liu Z Chen W Up-to-date comparison of robotic-assisted vs. open distal pancreatectomy: a PRISMA-compliant meta-analysis. Medicine (Baltimore). (2020) 99(23):e20435. 10.1097/md.0000000000020435 32501990 PMC7306371

[8] Müller PC Erdem S Kuemmerli C Nickel F Gehrisch OHF Uzunoglu FG International validation of the distal pancreatectomy fistula risk score: evaluation in minimally invasive and open surgery. Surg Endosc. (2025) 39(8):4863–71. 10.1007/s00464-025-11872-5 40542140 PMC12287223

[9] Zhou E Li X Zhao C Cui B. Comparison of perioperative and oncologic outcomes after open, laparoscopic, and robotic distal pancreatectomy: a single-center retrospective study. Updates Surg. (2024) 76(2):471–8. 10.1007/s13304-023-01658-6 37812318

[10] Nickel F Distler M Limen EF Wise PA Kowalewski KF Tritarelli PM Initial learning curves of laparoscopic and robotic distal pancreatectomy compared with open distal pancreatectomy: multicentre analysis. Br J Surg. (2023) 110(9):1063–7. 10.1093/bjs/znad045 36928918

[11] Chen W Lin X Wu Z Pan W Ke Q Chen Y. Laparoscopic liver resection is superior to radiofrequency ablation for small hepatocellular carcinoma: a systematic review and meta-analysis of propensity score-matched studies. Hepatol Int. (2024) 18(3):998–1010. 10.1007/s12072-024-10645-x 38480604

[12] Tang G Zhang L Xia L Zhang J Chen R Zhou R. Comparison of short-term outcomes of robotic vs. open pancreaticoduodenectomy: a meta-analysis of randomized controlled trials and propensity-score-matched studies. Int J Surg. (2025) 111(1):1214–30. 10.1097/js9.0000000000001871 38935118 PMC11745760

[13] Cucchetti A Bocchino A Crippa S Solaini L Partelli S Falconi M Advantages of laparoscopic distal pancreatectomy: systematic review and meta-analysis of randomized and matched studies. Surgery. (2023) 173(4):1023–9. 10.1016/j.surg.2022.11.029 36564287

[14] Magistri P Boggi U Esposito A Carrano FM Pesi B Ballarin R Robotic vs open distal pancreatectomy: a multi-institutional matched comparison analysis. J Hepatobiliary Pancreat Sci. (2021) 28(12):1098–106. 10.1002/jhbp.881 33314791

[15] Page MJ McKenzie JE Bossuyt PM Boutron I Hoffmann TC Mulrow CD The PRISMA 2020 statement: an updated guideline for reporting systematic reviews. Int J Surg. (2021) 88:105906. 10.1016/j.ijsu.2021.105906 33789826

[16] Higgins JP Thompson SG. Quantifying heterogeneity in a meta-analysis. Stat Med. (2002) 21(11):1539–58. 10.1002/sim.1186 12111919

[17] Weng Y Jin J Huo Z Shi Y Jiang Y Deng X Robotic-assisted vs. open distal pancreatectomy for benign and low-grade malignant pancreatic tumors: a propensity score-matched study. Surg Endosc. (2021) 35(5):2255–64. 10.1007/s00464-020-07639-9 32458287 PMC8057962

[18] Park EJ Noh GT Lee YJ Park MY Yang SY Han YD Robotic surgery may lead to reduced postoperative inflammatory stress in colon cancer: a propensity score-matched analysis. Ann Coloproctol. (2024) 40(6):594–601. 10.3393/ac.2024.00171.0024 39748552 PMC11701452

[19] Li R Zhou J Zhao S Sun L Fu Y Zhang C Propensity matched analysis of minimally invasive and open radical resection for rectal cancer: comparison of short-term outcomes in elderly/frail patients. J Robot Surg. (2024) 18(1):117. 10.1007/s11701-024-01883-0 38466495

[20] Manara M Aiolfi A Bonitta G Schlanger D Popa C Lombardo F Short-term outcomes analysis comparing open, lap-assisted, totally laparoscopic, and robotic total gastrectomy for gastric cancer: a network meta-analysis. Cancers (Basel). (2024) 16(19):3404. 10.3390/cancers16193404 39410024 PMC11475391

[21] de Rooij T van Hilst J van Santvoort H Boerma D van den Boezem P Daams F Minimally invasive vs. open distal pancreatectomy (LEOPARD): a multicenter patient-blinded randomized controlled trial. Ann Surg. (2019) 269(1):2–9. 10.1097/sla.0000000000002979 30080726

[22] van Bodegraven EA Francken MFG Verkoulen K Abu Hilal M Dijkgraaf MGW Besselink MG. Costs of complications following distal pancreatectomy: a systematic review. HPB (Oxford). (2023) 25(10):1145–50. 10.1016/j.hpb.2023.03.007 37391314

[23] Weinberg L Ratnasekara V Tran AT Kaldas P Neal-Williams T D’Silva MR The association of postoperative complications and hospital costs following distal pancreatectomy. Front Surg. (2022) 9:890518. 10.3389/fsurg.2022.890518 35711711 PMC9195500

[24] Korrel M Jones LR van Hilst J Balzano G Björnsson B Boggi U Minimally invasive vs. open distal pancreatectomy for resectable pancreatic cancer (DIPLOMA): an international randomised non-inferiority trial. Lancet Reg Health Eur. (2023) 31:100673. 10.1016/j.lanepe.2023.100673 37457332 PMC10339208

[25] Xu WY Xin J Yang Y Wang QW Yuan BH Peng FX. A comprehensive analysis of robotic assisted vs. laparoscopic distal pancreatectomy using propensity score matching. J Robot Surg. (2025) 19(1):86. 10.1007/s11701-025-02249-w 40014153

[26] Pitakteerabundit T Fagenholz PJ Luckhurst CM Srinivas Rao S Kambadakone A Warshaw AL Pancreatic fistula and intraabdominal fluid collections after distal pancreatectomy: incidence, implications, and natural history. Ann Surg. (2025). 10.1097/sla.0000000000006635 39829430

[27] Gavriilidis P Roberts KJ Sutcliffe RP. Comparison of robotic vs laparoscopic vs open distal pancreatectomy. A systematic review and network meta-analysis. HPB (Oxford). (2019) 21(10):1268–76. 10.1016/j.hpb.2019.04.010 31080086

[28] De Pastena M Paiella S Fontana M Filippini C Addari L Giorgi A The clinical and economic impact of surgical site infections after distal pancreatectomy. Surgery. (2022) 171(6):1652–7. 10.1016/j.surg.2021.11.010 34972593

[29] Lin ZY Zhang XP Zhao GD Li CG Wang ZH Liu R Short-term outcomes of robotic vs. open hepatectomy among overweight patients with hepatocellular carcinoma: a propensity score-matched study. BMC Surg. (2023) 23(1):153. 10.1186/s12893-023-02058-8 37286991 PMC10246414

[30] Simhal RK Simon DP Wang KR Shah YB Havranek B Mark JR Perioperative and complication related outcomes for robotic-assisted vs. open radical cystectomy: a comparative national surgical quality improvement project analysis. J Endourol. (2024) 38(4):331–9. 10.1089/end.2023.0279 38269428

[31] Chen Q Fu Y Li Y Cai H Wang X Wu Z Interim analysis of short-term outcomes after laparoscopic spleen-preserving distal pancreatectomy with or without preservation of splenic vessels: a randomised controlled trial. Int J Surg. (2025) 111(1):617–27. 10.1097/js9.0000000000001874 38954668 PMC11745598

[32] Korrel M Lof S Al Sarireh B Björnsson B Boggi U Butturini G Short-term outcomes after spleen-preserving minimally invasive distal pancreatectomy with or without preservation of splenic vessels: a Pan-European retrospective study in high-volume centers. Ann Surg. (2023) 277(1):e119–e25. 10.1097/sla.0000000000004963 34091515

[33] van Ramshorst TME van Bodegraven EA Zampedri P Kasai M Besselink MG Abu Hilal M. Robot-assisted vs. laparoscopic distal pancreatectomy: a systematic review and meta-analysis including patient subgroups. Surg Endosc. (2023) 37(6):4131–43. 10.1007/s00464-023-09894-y 36781467 PMC10235152

[34] Rompianesi G Montalti R Ambrosio L Troisi RI. Robotic vs. laparoscopic surgery for spleen-preserving distal pancreatectomies: systematic review and meta-analysis. J Pers Med. (2021) 11(6):552. 10.3390/jpm11060552 34199314 PMC8231987

[35] Mavrovounis G Diamantis A Perivoliotis K Symeonidis D Volakakis G Tepetes K. Laparoscopic vs. robotic peripheral pancreatectomy: a systematic review and meta-analysis. J Buon. (2020) 25(5):2456–75. 33277870

[36] Howard TJ Krug JE Yu J Zyromski NJ Schmidt CM Jacobson LE A margin-negative R0 resection accomplished with minimal postoperative complications is the surgeon’s contribution to long-term survival in pancreatic cancer. J Gastrointest Surg. (2006) 10(10):1338–45. 10.1016/j.gassur.2006.09.008 17175452

[37] Wang W Shen Z Zhang J Chen H Deng X Peng C A novel criterion for lymph nodes dissection in distal pancreatectomy for ductal adenocarcinoma: a population study of the US SEER database. Ann Surg Oncol. (2022) 29(3):1533–9. 10.1245/s10434-021-10797-2 34622372

[38] Kamarajah SK Sutandi N Sen G Hammond J Manas DM French JJ Comparative analysis of open, laparoscopic and robotic distal pancreatic resection: the United Kingdom’s first single-centre experience. J Minim Access Surg. (2022) 18(1):77–83. 10.4103/jmas.JMAS_163_20 35017396 PMC8830579

[39] De Pastena M Esposito A Paiella S Surci N Montagnini G Marchegiani G Cost-effectiveness and quality of life analysis of laparoscopic and robotic distal pancreatectomy: a propensity score-matched study. Surg Endosc. (2021) 35(3):1420–8. 10.1007/s00464-020-07528-1 32240383

[40] De Pastena M Esposito A Paiella S Montagnini G Zingaretti CC Ramera M Nationwide cost-effectiveness and quality of life analysis of minimally invasive distal pancreatectomy. Surg Endosc. (2024) 38(10):5881–90. 10.1007/s00464-024-10849-0 39164438 PMC11458716

[41] Magge DR Zenati MS Hamad A Rieser C Zureikat AH Zeh HJ Comprehensive comparative analysis of cost-effectiveness and perioperative outcomes between open, laparoscopic, and robotic distal pancreatectomy. HPB (Oxford). (2018) 20(12):1172–80. 10.1016/j.hpb.2018.05.014 31217087

[42] Koh YX Zhao Y Tan IE Tan HL Chua DW Loh WL Evaluating the economic efficiency of open, laparoscopic, and robotic distal pancreatectomy: an updated systematic review and network meta-analysis. Surg Endosc. (2024) 38(6):3035–51. 10.1007/s00464-024-10889-6 38777892

[43] Lof S Claassen L Hannink G Al-Sarireh B Björnsson B Boggi U Learning curves of minimally invasive distal pancreatectomy in experienced pancreatic centers. JAMA Surg. (2023) 158(9):927–33. 10.1001/jamasurg.2023.2279 37378968 PMC10308297

